# A Giant Dissecting Aneurysm of Ascending Aorta Following Aortic Valve Replacement

**DOI:** 10.1155/2014/541754

**Published:** 2014-04-15

**Authors:** Faruk Hökenek, Mete Gürsoy, Füsun Gülcan, Egemen Duygu, Murat Şener

**Affiliations:** ^1^Department of Cardiovascular Surgery, Aile Hospital, Acibadem University, Bahcelievler 34590 Istanbul, Turkey; ^2^Department of Anesthesiology, International Hospital, Acibadem University, Bahcelievler 34590 Istanbul, Turkey; ^3^Department of Cardiology, Aile Hospital, Acibadem University, Bahcelievler 34590 Istanbul, Turkey

## Abstract

Ascending aortic dissection and aneurysm are rare but life-threatening complications after aortic valve replacement. Preoperative evaluation of risk factors such as aortic diameter, structural features of aortic wall, and associated diseases may decrease complication rate. We herein present analysis of risk factors of proximal aortic events following aortic valve replacement based on patient with giant dissecting aneurysm who underwent modified Bentall procedure.

## 1. Introduction

Aortic dissection is infrequent but life-threatening complication after aortic valve replacement. The incidence of type A dissection varies between 0.2% and 2.3% [1, 2]. Although aortic valve replacement is considered as an independent risk factor for development of subsequent dissection, predisposing factors at the time of initial operation were well defined. Aortic regurgitation with systemic hypertension, male sex, thinned or fragile aortic wall, and ascending aortic dilatation were reported as a risk factor previously [3, 4]. In this paper, we present successful surgical treatment of giant dissecting aneurysm of ascending aorta late after aortic valve replacement and literature review.

## 2. Case Report

**Figure 1:**
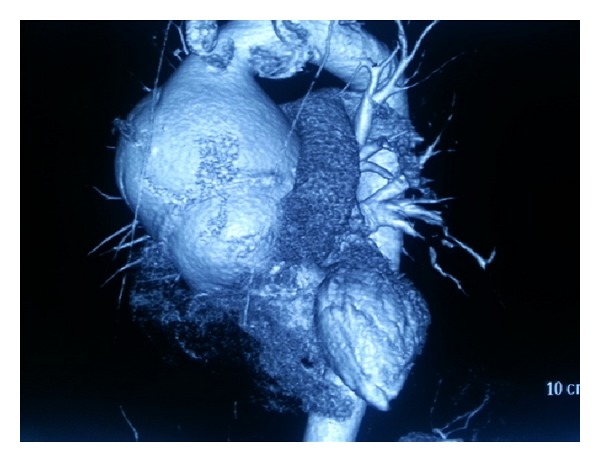
Computerized tomography shows ascending aortic aneurysm.

**Figure 2:**
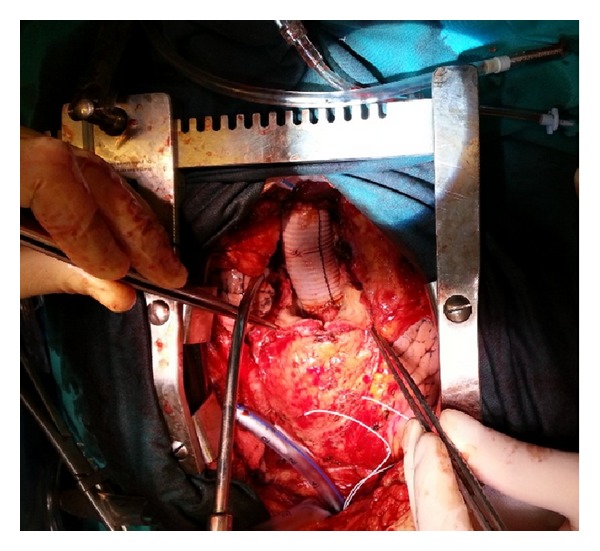
Postoperative view shows completed modified Bentall Procedure.

A 41-year-old woman was admitted to our institution with chest pain and dyspnea. Patient's history revealed aortic valve replacement taking place two years ago, patient's native valve was tricuspid and ascending aorta diameter was 4.3 cm at the time of operation. Physical examination revealed systolic 3/6 murmur along the left sternal border. Transthoracic echocardiography showed functional prosthetic aortic valve and ascending aortic aneurysm including sinuses of valsalva with dissection flap extending to the innominate artery. Aneurysm's transverse diameter was measured as 8.5 cm with computerized tomography (Figure 1). Coronary arteries were found normal by angiography. Patient underwent redo cardiac surgery. We institutioned femorofemoral cardiopulmonary bypass and reopened sternum. A giant ascending aorta aneurysm was seen. Following retrograde cold blood cardioplegia, aneurysm was transected just above the right coronary artery ostium. Prosthetic valve was functional but supra annular space was considered inappropriate to graft interposition. Prosthetic valve was removed. Modified Bentall procedure was performed with no: 25 valved conduit. Distal anastomosis was done under aortic cross clamp. Operation was completed as usual fashion (Figure 2). Postoperative course was uneventful. Patient was discharged at the postoperative 8th day in well condition.

## 3. Discussion

Proximal aortic events including dissection, aneurysm, and rupture are rare but potentially fatal complications following aortic valve replacement. Although indications of simultaneous ascending aorta and aortic valve replacement are well established, patients in gray zone remain to be a challenge for surgeons. In this case patients had undergone the aortic valve replacement for severe aortic regurgitation. Patients preoperative ascending aorta diameter had been measured as 43 mm and aortic valve had been found tricuspid and myxomatous with echocardiography. Although patients ascending aorta diameter at the time of valve replacement had been considered below indicated limits of surgery, several reports have been published regarding aortic dissection in such patients [5, 6].

In patients with thoracic aorta aneurysm, decision making depends on the diameter of aneurysm, concomitant diseases, and structural features of aortic wall. Recommended indications for elective replacement of the aorta are summarized in Table 1 [7].

Maximum aneurysm diameter directly correlates with mortality from aortic rupture and dissection when the patients aneurysm diameter reaches ≥44 mm. At diameters greater than 60 mm, the annual rate of rupture and dissection is about 6.9% and mortality 11.8%. Complication rate increases around twofold in comparison with patients with an aortic diameter of 50 mm to 59 mm but this increase is lower in smaller sizes (10% to 25% per 10 mm) [8, 9]. On the other hand, genetic disorders affecting connective tissue including Marfan syndrome, vascular Ehlers-Danlos syndrome, Turner syndrome, and Loeys-Dietz syndrome are strongly associated with increased risk of aneurysm expansion, dissection, and rupture [9]. Recent reports recommend close cardiologic surveillance when aortic aneurysm size index >2.0 cm/m^2^. Patients with aforementioned genetic diseases are at the highest risk when aortic size index ≥2.5 cm/m^2^ [10–12]. Besides diameter, growth rate of aneurysm (5 mm/year) is accepted indication for surgery [7].

Aortic valve pathology causing valve replacement or repair may be efficacious in development of aneurysm. Benedik et al. reported that patients with aortic regurgitation have poorer quality of the ascending aortic wall compared with patients with aortic stenosis at the time of aortic valve replacement. Their study showed that patients presenting for aortic valve replacement with AR have higher tendency to ascending aorta aneurysm development (≥50 mm) than patients with aortic stenosis in followup [13].

Bicuspid aortic valve (BAV) is the most common congenital heart disease which causes aortic stenosis, regurgitation, aortic aneurysm, and dissection. Tzemos et al. evaluated prognosis of bicuspid aortic valve. In their series, 142 (22%) of 642 patients experienced aortic valve and/or root surgery and aortic dissection and aneurysm occurred in 2 percent of patients in followup [14]. Cardiac complication risk of patients with bicuspid aortic valve is significantly higher than that of general population. Although proximal aortic events rate is increased after AVR in patients with bicuspid valve, dissection and cardiac mortality risk are similar with patients with tricuspid valve [7, 15]. Prophylactic aortic replacement should be considered in patients with BAV when aortic size ≥50 mm particularly in the presence of additional risk factors [1, 7, 15].

Although male gender was found to be independent risk factor of proximal aortic events after AVR, it has not been used as decisive parameter for surgical indication [3]. Relatively lower body surface area of women may necessitate the use of indexed values in risk prediction. On the other hand, intention to have children may be considered relative indication for simultaneous aortic root replacement in women with Marfan or Loeys-Dietz syndrome [7].

Hypertension should be regulated precisely following AVR especially in patients with aortic dilatation. The aim of medical therapy is to reduce wall stress in patients with aortic aneurysm. Beta blockers have proven to have effect in prevention of aneurysm progression so they should be utilized in each patient with hypertension. In our patient's history, unregulated hypertension was significant which may be triggering factor of dissecting aneurysm.

While guidelines are data driven, the surgeon's expertise and each individual patient's case determine the ultimate management decision as occurred in our case.

## 4. Conclusion

Individual decision making depends on several parameters in borderline patients. Not only aortic diameter but also aortic wall quality, preoperative etiology, associated diseases, connective tissue disorders, genetic syndromes, congenital valve defects, and even sex should be consulted.

## Figures and Tables

**Table 1:** Recommended indications for elective replacement of the aorta.

Indication for elective surgical replacement of the aorta	Class of recommendation	Level of evidence
≥50 mm for patients with Marfan syndrome and connective tissue disorders	I	C
≥45 mm for patients with Marfan syndrome and risk factor*	IIa	C
≥50 mm for patients with bicuspid valve and risk factor*	IIa	C
≥55 mm for all other patients	IIa	C

*Risk factors: familial predisposition to aortic dissection, aneurysm growth rate > 5 mm/year, aortic valve morphology (unicuspid, bicuspid), corrected or uncorrected aortic coarctation, and intention to have children (female patients with Marfan or Loeys-Dietz Syndrome).
